# Leptin and insulin signaling in dopaminergic neurons: relationship between energy balance and reward system

**DOI:** 10.3389/fpsyg.2014.00846

**Published:** 2014-08-07

**Authors:** Doan V. Khanh, Yun-Hee Choi, Sang Hyun Moh, Ann W. Kinyua, Ki Woo Kim

**Affiliations:** ^1^Departments of Pharmacology and Global Biomedical Science, Wonju College of Medicine, Yonsei UniversityWonju, South Korea; ^2^Institute of Lifestyle Medicine and Nuclear Receptor Research Consortium, Wonju College of Medicine, Yonsei UniversityWonju, South Korea; ^3^Antiaging Research Institute of BIO-FD&C Co. Ltd.Incheon, South Korea

**Keywords:** leptin, insulin, midbrain, dopamine, reward, energy homeostasis

## Abstract

The central actions of leptin and insulin are essential for the regulation of energy and glucose homeostasis. In addition to the crucial effects on the hypothalamus, emerging evidence suggests that the leptin and insulin signaling can act on other brain regions to mediate the reward value of nutrients. Recent studies have indicated the midbrain dopaminergic neurons as a potential site for leptin’ and insulin’s actions on mediating the feeding behaviors and therefore affecting the energy balance. Although molecular details about the integrative roles of leptin and insulin in this subset of neurons remain to be investigated, substantial body of evidence by far imply that the signaling pathways regulated by leptin and insulin may play an essential role in the regulation of energy balance through the control of food-associated reward. This review therefore describes the convergence of energy regulation and reward system, particularly focusing on leptin and insulin signaling in the midbrain dopaminergic neurons.

## INTRODUCTION

Obesity, a multifactorial metabolic disorder that leads to many adverse health consequences, has reached epidemic proportions globally with more than 500 million adults being obese as of 2008 (Frühbeck et al., 2013). Obesity occurs as a result of genetic, behavioral, environmental, physiological, social, and cultural factors. Among the listed causes, behavioral and environmental factors have been described as the major contributors to the dramatic increase in obesity in the past two decades. The fundamental etiology of obesity is an energy imbalance between calorie consumption and energy expenditure with relatively higher food consumption (Racette et al., 2003; Nguyen and El-Serag, 2010). Drive for food consumption is a multiple process which is not only caused by nutritional status of the body but is also affected by the food palatability (the rewarding aspect of food) and other environmental and social factors. Increased energy intake due to excessive consumption of palatable food has contributed to the rise of obesity. It is well established that the hypothalamus plays a central role in regulation of energy balance and food intake to maintain the body’s physiological requirements. An extensive body of evidence has demonstrated that endocrine regulators such as insulin and leptin mainly act on the hypothalamus of the central nervous system (CNS) to regulate food intake and body weight. In addition, expression of leptin and insulin receptors in other regions of the brain such as the doparminergic (DA) neurons suggests that the two hormones exert their effects in other areas outside of the hypothalamus. The neuronal circuit of DA neurons mediating reward, motivational and hedonic mechanisms in the CNS is also involved in the regulation of many aspects of feeding behavior and energy homeostasis. Indeed, accumulating evidence has indicated that leptin and insulin act on the midbrain DA neurons mediating feeding behaviors and therefore affecting energy balance (Fulton et al., 2006; Homme et al., 2006; Figlewicz et al., 2008; Leinninger et al., 2009, 2011; Morton et al., 2009; Opland et al., 2010; Bruijnzeel et al., 2011; Domingos et al., 2011; Mebel et al., 2012). In this review, we seek to focus on the energy homeostasis role of insulin and leptin particularly in the midbrain DA reward circuit system.

## INSULIN AND LEPTIN IN CONTROL OF ENERGY BALANCE IN CNS

Studies on the infusion of insulin into the brain have opened the view that peripheral hormones can act on the brain to regulate food intake and body weight (Woods et al., 1979; Porte and Woods, 1981; Brief and Davis, 1984; Schwartz et al., 1992; Chavez et al., 1995, 1996; Air et al., 2002). Leptin, the adipose-derived hormone, was identified in the mid-1990 and it was shown to exert its actions mainly in the CNS (Zhang et al., 1994; Halaas et al., 1995). Since then, various studies have been carried out to elucidate the role of leptin in energy homeostasis particularly in the brain giving further insight into its role in obesity. Moreover, expression of insulin and leptin receptors throughout the brain confirmed, at least partially, the functional signaling of these hormones in the CNS (Havrankova et al., 1978; Gammeltoft et al., 1984; Zahniser et al., 1984; Werther et al., 1987; Unger et al., 1991; Kar et al., 1993; Huang et al., 1996; Couce et al., 1997; De Matteis and Cinti, 1998; Elmquist et al., 1998; Shioda et al., 1998; Burguera et al., 2000; Funahashi et al., 2003; Leshan et al., 2006). Various studies have also demonstrated the role of insulin and leptin signaling on glucose homeostasis in the brain. These studies employed different experimental models such as insulin receptor knock out and db/db mice, and Zucker fa/fa rats which lack leptin receptors in both CNS and periphery (Chua et al., 1996; Bruning et al., 2000; Koch et al., 2008). In addition neuron-specific leptin receptor knockout mice provided obvious evidence on the role of leptin action in the CNS (Balthasar et al., 2004; van de Wall et al., 2008).

The hypothalamic nuclei where both insulin and leptin receptors are strongly and widely expressed have been described as the key site for insulin and leptin actions in the CNS (McGowan et al., 1992; Satoh et al., 1997; Ring and Zeltser, 2010). It has been suggested that both insulin and leptin act on two functionally opposite groups of neurons in the arcuate nucleus (ARC) of the hypothalamus to provide negative feedback for food intake and energy balance. Leptin and insulin inhibit orexigenic neurons expressing neuropeptide Y (NPY)/agouti-related protein (AgRP), neuropeptides that are known to stimulate food intake and reduce energy expenditure. Conversely, they activate pro-opiomelanocortin (POMC)/cocaine and amphetamine related transcript (CART) neurons. Anorexic neurons expressing POMC, a protein precursor which is processed to melanocortins including α-melanocyte stimulating hormone (α-MSH), reduce food intake and increase energy expenditure (Schwartz et al., 2000; Morton et al., 2006; Belgardt and Bruning, 2010; Figlewicz and Sipols, 2010).

Other hypothalamic nuclei such as paraventricular nucleus (PVN) and lateral hypothalamic area (LHA) may directly or indirectly mediate the effects of insulin and leptin since these regions receive innervations from both NPY/AgRP and POMC/CART neurons and also express insulin and leptin receptors. The melanocortin receptors 3 and 4 (MC3/4R) and NPY receptors which respond to the anorexigenic effects of α-MSH and the orexigenic effects of NPY/AgRP, respectively, are expressed abundantly in the PVN and LHA (Mountjoy et al., 1994; Parker and Herzog, 1999). In addition, these neurons project to other brain regions that mediate the perception of satiety (e.g., the nucleus of the solitary tract, NTS, in the hindbrain) and the reward system (the mesolimbic DA system; Morton et al., 2006, for review). Recent studies showed that neurotensins-containing neurons in the LHA innervate to the local orexin neurons and the ventral tegmental area (VTA) of the DA system (Leinninger et al., 2011). Leptin was shown to act on the leptin receptor-expressing neurons in the LHA to control orexin and the mesolimbic DA system and contribute to the control of energy balance (Leinninger et al., 2009, 2011).

## INSULIN AND LEPTIN ACTIONS ON THE REWARD SYSTEM TO MODULATE ENERGY HOMEOSTASIS

The broad expression of insulin and leptin receptors in several CNS regions raised the question about their functions beyond the hypothalamus (Havrankova et al., 1978; Unger et al., 1991; Huang et al., 1996; Guan et al., 1997; Elmquist et al., 1998; Figlewicz et al., 2003; Funahashi et al., 2003; Fulton et al., 2006; Homme et al., 2006). Among these regions, the DA neuron system, which plays an important role in the regulation of reward and motivational behaviors, emerged as a potential target for insulin and leptin actions. The mesolimbic DA neurons project from the VTA and substantia nigra (SN) to the nucleus accumbens (NAc) and have been implicated in the rewarding and motivating aspects of food intake (Berridge, 1996; Saper et al., 2002; Kelley et al., 2005b; Wise, 2006). One of the factors contributing to increased incidences of obesity is diet composition especially in this modern era where most people opt for processed or instant foods. Given that the reward system directly or indirectly regulates feeding behaviors, there is therefore an increased interest in studies focusing on the role of the reward circuit and the DA neurons in modulating feeding behaviors and energy homeostasis (Figlewicz and Sipols, 2010).

Intra-cerebroventricular insulin injection decreased lever rates for sucrose solution, decreased sucrose self-administration (Figlewicz et al., 2006, 2008) and reversed conditioned place preference (CPP) with high fat diet (Figlewicz et al., 2004). CPP measures the ability of an animal to respond to the rewarding aspects of food and reduced CPP by insulin hence suggests that this hormone can modulate reward-related feeding behavior (Palmiter, 2007). Specifically, a recent study showed that direct administration of insulin into the VTA reduced food intake and repressed feeding of sweetened high-fat diet in the sated condition (hedonic feeding; Bruijnzeel et al., 2011; Mebel et al., 2012). Importantly, deletion of the insulin signaling in the catecholaminergic neurons resulted in increased sucrose sensitivity and an obese phenotype (Könner et al., 2011). However, catecholaminergic neurons represent both the dopaminergic and the norepinephrinergic neurotransmitter activities. Therefore, the exact mechanism underlying the effect of insulin signaling on hedonic and reward feeding behavior cannot be deduced solely from the observations made in catecholaminergic neurons. In an attempt to identify the mechanism of insulin signaling in DA system, the dopamine re-uptake transporter (DAT) has emerged as a potential cellular target for insulin action. DAT transports DA from the synapse back to the nerve terminal, hence decreasing dopamine activity (Jaber et al., 1997). Insulin increased DAT mRNA level and activity, this could lead to enhanced clearance of dopamine from the synapse and therefore reducing DA signaling (Figlewicz and Sipols, 2010, for review). To gain further mechanistic insight into the effect of insulin on the DA signaling, there is need to carry out more studies using an experimental model in which the insulin signaling has been disrupted specifically in the DA neurons.

Pharmacological studies have indicated that leptin also modulates behaviors associated with dopamine reward circuit. Leptin decreased lateral hypothalamic self-stimulation as well as sucrose self-administration and sucrose CPP (Figlewicz et al., 2001, 2004, 2006; Shalev et al., 2001). Moreover, leptin declined drug seeking behaviors caused by food deprivation (Shalev et al., 2001; Hao et al., 2004). In addition, direct leptin injection into the VTA reduced food intake (Homme et al., 2006; Morton et al., 2009; Bruijnzeel et al., 2011). These findings imply that leptin provides negative effects on DA reward neurons resulting in food intake reduction. Further evidence shows that leptin suppressed the mesolimbic DA signaling by decreasing the DA neuronal firing frequency and subsequently reducing DA levels in the NAc (Krügel et al., 2003; Homme et al., 2006). In addition, presynaptic leptin action can suppress excitatory synaptic transmission into DA neurons in VTA (Thompson and Borgland, 2013). Moreover, similar to the effects observed in insulin signaling, decreased DA concentrations by leptin could be attributed to increased DAT activity (Perry et al., 2010). However, investigations on the function of mesolimbic DA system in leptin-deficient animals showed opposite findings in that the DA signaling originating from the VTA was reduced. *Ob/ob* mice contained less tyrosine hydroxylase, the rate-limiting enzyme for DA synthesis, and showed decreased DA content in the VTA and NAc (Fulton et al., 2006; Roseberry et al., 2007). In addition, dopamine 2 (D2) receptor binding decreased in the VTA of these mice and this was reversed by leptin treatment (Pfaﬄy et al., 2010). It is difficult to explain the discrepancies observed in *ob/ob* mice but it is possible that chronic leptin deficiency stimulates other compensatory mechanisms, for example, chronic leptin deficiency might lead to changes in normal intracellular signaling pathways and activate a feedback regulatory loop that might be responsible for regulating DA content and function and ultimately decreasing the function of DA neurons (Opland et al., 2010, for review).

Genetic techniques using viral-mediated RNA to knock down the leptin receptor in the VTA also showed increase in food intake and sensitivity for highly palatable food highly suggesting the crucial role of leptin in VTA in modulation of feeding behavior and energy homeostasis (Homme et al., 2006; Davis et al., 2011). Consistently, recent studies using optogenetic approach to activate DA neurons and quantify the reward value of nutrients strongly confirmed the negative effects of leptin on the reward value via reduction in DA signaling (Domingos et al., 2011). However, DA neurons-specific knockdown of leptin receptor using cre-loxP system (Lepr^DAT-Cre^) showed no change in body weight or food intake (Liu et al., 2011). This could be because disrupting the leptin signaling only in a small subset of leptin receptor-expressing neurons in VTA in Lepr^DAT-Cre^ mice might not be sufficient to affect energy balance and this loss might be compensated by other leptin receptor neurons in the other brain regions. Therefore, further investigation using different genetic approaches with higher sensitivity such as tissue-specific re-activation of leptin receptor signaling only in dopaminergic neurons might be helpful to assess the role of leptin receptor signaling in this reward circuit.

As mentioned above, LHA has been suggested as a target for leptin action to modulate the reward circuit. In addition, it has been suggested that the group of neuron in the LHA project to the mesolimbic regions to control DA action and reward (DiLeone et al., 2003; Harris et al., 2005; Kelley et al., 2005a; Opland et al., 2010). Among these, two populations of neurons have been identified: melanin concentrating hormone (MCH) and orexin expressing neurons. These neurons are known to project to the NAc and VTA, respectively, to promote feeding and modulate reward (Qu et al., 1996; Mieda and Yanagisawa, 2002; Georgescu et al., 2005). However, leptin is known to inhibit orexin and MCH activities in this circuitry (Qu et al., 1996; Yamanaka et al., 2003). Interestingly, LHA also consists of neurons expressing leptin receptors which are distinct from MCH and orexin neurons and innervate to the VTA. Moreover, leptin acts on these neurons to modulate the mesolimbic DA system and decrease feeding (Leinninger et al., 2009). Recent studies demonstrated that majority of leptin receptor neurons in LHA contain neurotensins (Nts) and leptin receptors in Nts neurons project to the VTA and local orexin neurons but not MCH neurons to mediate the physiological action of leptin on orexin neurons and the mesolimbic DA system (Leinninger et al., 2011).

## INSULIN AND LEPTIN SIGNALING PATHWAYS IN CNS

**FIGURE 1:**
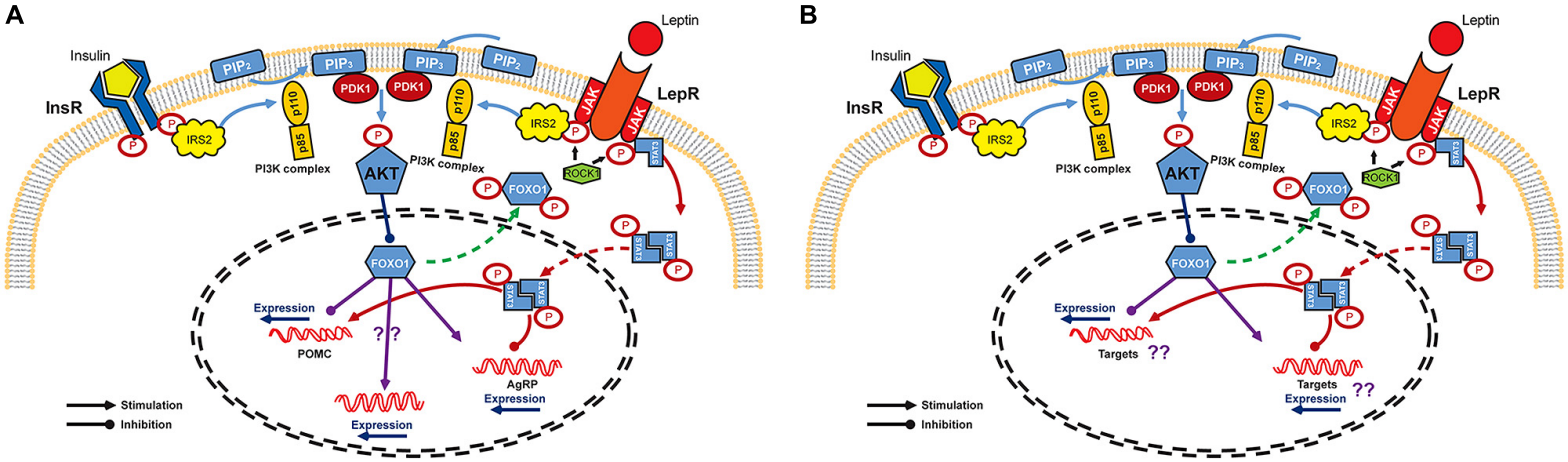
**Signaling pathways of leptin and insulin in the CNS. (A)** Leptin and insulin pathways converge on IRS/PI3K/Akt axis in arcuate nucleus of the hypothalamus. Binding of insulin and leptin to their receptors leads to the phosphorylation of IRS2 which activates PI3K to facilitate the phosphorylation of Akt by PDK. Akt activation finally phosphorylates and excludes FoxO1 from nucleus to inactivate it. FoxO1 is known as a repressor of POMC expression, but as an activator of AgRP expression. Expression of POMC and AgRP is also regulated by JAK/STAT3 pathway. Once leptin binds to its receptor, STAT3 is phosphorylated by JAK and is dimerized. The active dimeric form of STAT3 translocates to the nucleus where it inhibits AgRP and activates POMC expression. **(B)** Schematic diagram of insulin and leptin signaling in DA neurons. InR, insulin receptor; LepR, leptin receptor; IRS, insulin receptor substrate; PIP2, phosphatidylinositol-4,5-biphosphate; ROCK1, Rho-kinase 1; PIP3, phosphatidylinositol-3,4,5-triphosphate; PI3K, phosphatidylinositol 3-OH kinase; PDK1, phosphoinositide-dependent kinase-1; Akt, protein kinase B; FoxO1, forkhead transcription factor O1; JAK, Janus kinase; STAT3, signal transducer and activator of transcription 3; POMC, proopiomelanocortin; AgRP, agouti-related protein.

Studies in the hypothalamus have provided a basis for understanding the molecular mechanism of insulin and leptin in the CNS even though the entire mechanism remains to be elucidated. The CNS insulin signaling is quite similar to that in peripheral organs. Insulin binds to and activates its receptor, a tyrosine kinase with autophosphorylating activity, and receptor activation leads to phosphorylation of insulin receptor substrate (IRS)/phosphatidylinositol 3-OH kinase (PI3K) pathway (Hadari et al., 1992). The catalytic subunit p110 of PI3K complex converts phosphatidylinositol-4,5-biphosphate (PIP2) into phosphatidylinositol-3,4,5-triphosphate (PIP3) to phosphorylate and activate downstream Akt/PKB (Niswender et al., 2003). This Akt activation in turn phosphorylates forkhead transcription factor O1 (FoxO1) which functions, especially in ARC, as a transcriptional suppressor of POMC gene and as a transcriptional activator of AgRP gene (Kitamura et al., 2006; Ren et al., 2012). Phosphorylated form of FoxO1 is subsequently excluded to the cytoplasm, allowing binding of transcriptional stimulators such as pSTAT3 to the POMC promoter. At the same time, FoxO1-mediated AgRP expression is inhibited (Plum et al., 2006; Varela and Horvath, 2012, for reviews; **Figure 1A**). However, unlike the peripheral insulin signaling in which IRS1 protein plays an essential role in insulin signal transduction, it has been suggested that the IRS2 is a major player involved in CNS insulin action on energy homeostasis regulation (Davis et al., 2004; Kubota et al., 2004; Porte et al., 2005, for review). IRS1 is sparsely expressed in the ventral hypothalamus and IRS1-deficient mice do not express abnormal metabolic phenotype (Araki et al., 1994; Tamemoto et al., 1994). On the other hand, IRS2 is abundant in the ARC and tyrosine phosphorylation of IRS2 is associated with increased PIP3, indicating the activation of PI3K/Akt downstream pathway mainly through IRS2 (Niswender and Schwartz, 2003; Torsoni et al., 2003). In support of this notion, mice lacking IRS2 showed increased food intake and fat mass and impaired reproductive activity (Burks et al., 2000).

Leptin binding to its receptor triggers IRS phosphorylation and also activate PI3K activity (Niswender and Schwartz, 2003). However, leptin receptor does not have intrinsic tyrosine kinase activity and requires JAK-STAT binding for full activation (Sweeney, 2002, for review). Leptin binding to its receptor allows JAKs in juxtaposition to phosphorylate and activate each other. In addition, recent report revealed that Rho-kinase 1 (ROCK1) plays a critical role in leptin signaling by phosphorylating JAK2 via a direct ROCK1-JAK2 interaction (Huang et al., 2012; **Figure 1A**). Phosphorylation of leptin receptor allows association of STAT, a substrate for JAK. After its dissociation form leptin receptor, STAT is phosphorylated and forms active dimers. Activated pSTAT3 translocates to the nucleus leading to transcriptional events such as stimulating POMC and inhibiting AgRP expression (Leshan et al., 2006; Mesaros et al., 2008; Ernst et al., 2009; **Figure 1**).

Although leptin and insulin mediate somewhat independent neuronal responses, there seems to be a crosstalk between these two hormones in energy homeostasis in the CNS (Niswender and Schwartz, 2003; Benomar et al., 2005; Porte et al., 2005). Specifically, it has been demonstrated that the IRS/PI3K/Akt axis is important for both insulin and leptin action in CNS (Niswender et al., 2003; Xu et al., 2005, 2010; Hill et al., 2008; **Figure 1**). Moreover, this overlap might also exist in the molecular pathways that provide negative effects to the insulin and leptin signaling such as the phosphatase protein tyrosine phosphatase 1B (PTP1B) and the suppressor of cytokine signaling 3 (SOCS3). PTP1B inhibits both insulin and leptin signaling and mice lacking PTP1B are more sensitive to both leptin and insulin and resistant to diet-induced obesity (Elchebly et al., 2000; Zabolotny et al., 2002). SOCS3 is a known negative regulator of leptin (cytokine in general) signaling (Sweeney, 2002). However, SOCS3 can also cause insulin resistance by modifying insulin receptor and IRS proteins leading to impaired insulin signaling (Rieusset et al., 2004; Ueki et al., 2004). The FoxO1, a nuclear transcriptional factor downstream of the PI3K/Akt axis which is known to mediate insulin action, might also be a potential crosstalk in the insulin and leptin signaling (Altomonte et al., 2004; Barthel et al., 2005). A recent study pointed out the crucial role of FoxO1 in the mediation of IRS2/PI3K signaling in LepR-expressing neurons to control energy balance (Sadagurski et al., 2012). The functional signaling of insulin and leptin, together with the presence of insulin and leptin receptors, have been confirmed in the VTA (Fulton et al., 2006; Homme et al., 2006; Iñiguez et al., 2008). PI3K activity is increased under direct administration of insulin and leptin into the VTA (Figlewicz et al., 2007). Moreover, IRS2/Akt pathway in VTA has been shown to modulate rewarding and psychomotor activating effects of cocaine and opiates (Russo et al., 2007; Iñiguez et al., 2008). Direct leptin administration into the VTA increased JAK-STAT signaling and this is essential for the effect of leptin in the VTA to decrease food intake (Morton et al., 2009). Therefore, studies on the molecular crosstalk occurring downstream of leptin and insulin in DA neurons may also be important to understand specific roles of these signals in mediating energy homeostasis and reward value of food (**Figure 1B**).

## CONCLUSION

Taken together, both pharmacological and genetic studies demonstrate that insulin and leptin not only act on hypothalamic regions but also play important roles in the DA reward system to regulate feeding behavior and energy balance. Further, leptin and insulin in DA neurons seem to mediate several neuronal projections to the other brain regions such as hypothalamus and NAc that are potentially important for the regulation of feeding and mood behaviors. At a cellular level, establishing whether leptin and insulin act on the same or different populations of DA neurons would be important to distinguish their specific functions in the DA neurons and in other neuronal projections. Therefore, studies using more advanced techniques such as optogenetics and pharmacogenetic tools will be beneficial to further understand the neuronal and molecular mechanisms underlying the effects of insulin and leptin on this reward system.

## AUTHOR CONTRIBUTIONS

Doan V. Khanh: drafted and edited the manuscript. Yun-Hee Choi, Ann W. Kinyua and Sang Hyun Moh: reviewed the manuscript and finalized figure. Ki Woo Kim: drafted, edited and finalized the manuscript.

## Conflict of Interest Statement

The authors declare that the research was conducted in the absence of any commercial or financial relationships that could be construed as a potential conflict of interest.
